# Magnetic Network on Demand: Pressure Tunes Square Lattice Coordination Polymers Based on {[Cu(pyrazine)_2_]^2+^}_*n*_

**DOI:** 10.1021/acs.inorgchem.0c01229

**Published:** 2020-07-03

**Authors:** Rebecca Scatena, Fabio Montisci, Arianna Lanza, Nicola P. M. Casati, Piero Macchi

**Affiliations:** † Department of Chemistry and Biochemistry, University of Bern, Freiestrasse 3, 3012 Bern, Switzerland; ‡ Center for Nanotechnology Innovation @NEST, Istituto Italiano di Tecnologia, Piazza San Silvestro 12, 56127 Pisa, Italy; § Paul Scherrer Institute, Laboratory for Synchrotron Radiation Condensed Matter, Forschungsstrasse 111, 5232 Villigen, Switzerland; ∥ Department of Chemistry, Materials and Chemical Engineering, Polytechnic of Milan, via Mancinelli 7, 20131 Milan, Italy

## Abstract

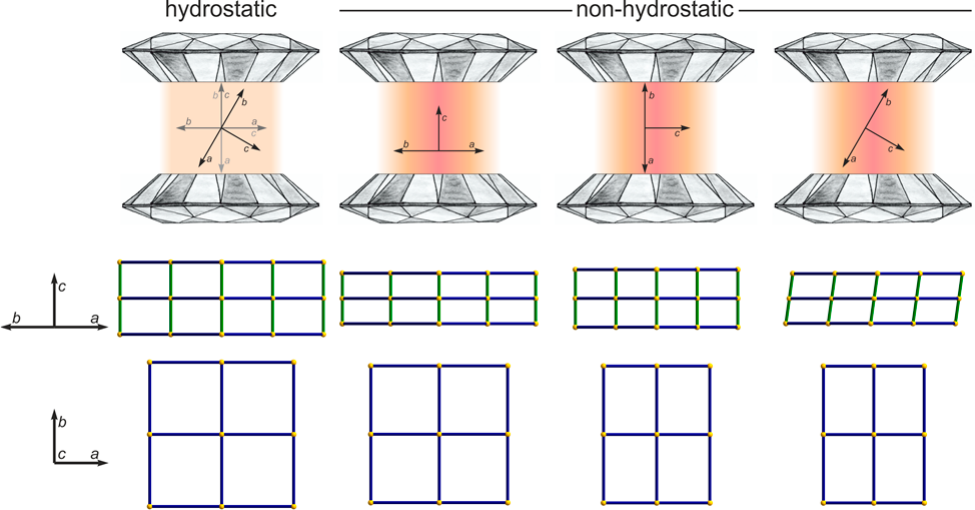

We report the pressure-induced structural and magnetic changes in [CuCl(pyz)_2_](BF_4_) (pyz = pyrazine) and [CuBr(pyz)_2_](BF_4_), two members of a family of three-dimensional coordination polymers based on square mesh {[Cu(pyz)_2_]^2+^}_*n*_ layers. High-pressure X-ray diffraction and density functional theory calculations have been used to investigate the structure–magnetic property relationship. Although structurally robust and almost undeformed within a large pressure range, the {[Cu(pyz)_2_]^2+^}_*n*_ network can be electronically modified by adjusting the interaction of the apical linkers interconnecting the layers, which has strong implications for the magnetic properties. It is then demonstrated that the degree of covalent character of the apical interaction explains the difference in magnetic exchange between the two species. We have also investigated the mechanical deformation of the network induced by nonhydrostatic compression that affects the structure depending on the crystal orientation. The obtained results suggest the existence of “Jahn–Teller frustration” triggered at the highest hydrostatic pressure limit.

## Abstract 2

Structural deformations imply changes in the properties of materials. The tetragonal framework of [CuX(pyrazine)_2_](BF_4_) (X = Cl or Br) coordination polymers can be deformed by compression, which causes modifications of their electronic structure. The magnitude of the antiferromagnetic coupling in the two-dimensional [Cu(pyrazine)_2_]^2+^ network is found to be particularly sensitive to the compression of the perpendicular Cu−X interaction, allowing the material to transform from a quasi-two-dimensional to a three-dimensional magnet via the effect of pressure alone.

## Introduction

Coordination polymers, in which metal ions (nodes) are connected via organic ligands (linkers) and stabilized by extra-framework inorganic anions, are ideal platforms for the investigation of low-dimensional quantum magnetism.^1,2^ A special interest in this class of materials concerns the emerging field of spintronics, in which scientists exploit the electron spin (instead of the electron charge) as an information carrier.^3^ Progress in this field requires a deep understanding of fundamental chemical and magnetic interactions in solid-state materials. There are several reasons to explore coordination polymers in this context. (a) Their properties can be fine-tuned by taking advantage of the rich functionality of the organic ligand. (b) They are typical charge transfer insulators. (c) Their magnetism is determined by the spin moments of the metals, which are mainly antiferromagnetically ordered and localized at the nodes.^4−6^ (d) They allow a high degree of topological control of the properties because coordination polymers are easily produced in crystalline form. Moreover, the relatively soft nature of these hybrid organic–inorganic materials provides the opportunity to tweak the spin behavior using external stimuli, potentially leading to multifunctional spintronic devices.^7,8^ Physical responses to stimuli require atomic rearrangement in the solid-state structure; the application of pressure to low-dimensional systems is a promising strategy for achieving this goal.^9−11^ Pressure also represents a terrific tool for probing the changes in the properties while modifying the structure in a controlled way, thus allowing for a deep understanding of structure–property relationships.

In this work, we focus on a family of three-dimensional (3D) coordination polymers based on square mesh {[Cu(pyz)_2_]^2+^}_*n*_ cationic layers (pyz = pyrazine), connected by inorganic X^–^ ligands and stabilized by extra-framework Y^–^ anions, where, for instance, X^–^ = HF_2_^–^, NO_3_^–^, BF_4_^–^, ClO_4_^–^, or NO_2_^–^ and Y^–^ = NO_3_^–^, BF_4_^–^, ClO_4_^–^, SbF_6_^–^, TaF_6_^–^, AsF_6_^–^, or PF_6_^–^. Within this family, the Cu–pyz–Cu pathway forming the {[Cu(pyz)_2_]^2+^}_*n*_ network is responsible for the strongest magnetic interaction in the system, which is antiferromagnetic (AFM) in nature and infers quasi-two-dimensional magnetic properties.^12−17^ These two-dimensional (2D) properties are well justified by the Cu^II^ coordination environment that suffers from pseudo-Jahn–Teller (JT) distortion and sets the equatorial plane with the singularly occupied magnetic orbital on the [Cu(pyz)_2_]^2+^ plane.^12,18^

Goddard et al.^17^ pointed out that, despite the structural similarity of the {[Cu(pyz)_2_]^2+^}_*n*_ network within the family of polymers, the AFM coupling through pyz (*J*_pyz_) can easily vary from <5 to >15 K. This means that there is a weak coupling but it is associated with a large variability, up to 300%. To explain this, it has been hypothesized that the tilting angle of pyz with respect to the equatorial plane of Cu^II^ octahedra may tune the overlap between the magnetic orbital at the metal and the delocalized π-electron system of the aromatic pyz ring. However, more recent studies of the accurate charge density distribution in some of these materials have shown that the role of the π-electron system of pyz is hampered and that the magnetic superexchange occurring through pyz is σ-driven.^12,18^ The same studies also showed that the extra-framework anions make purely closed-shell, ionic bonds to the networks, excluding their participation in the magnetic superexchange mechanism.

**Figure 1:**
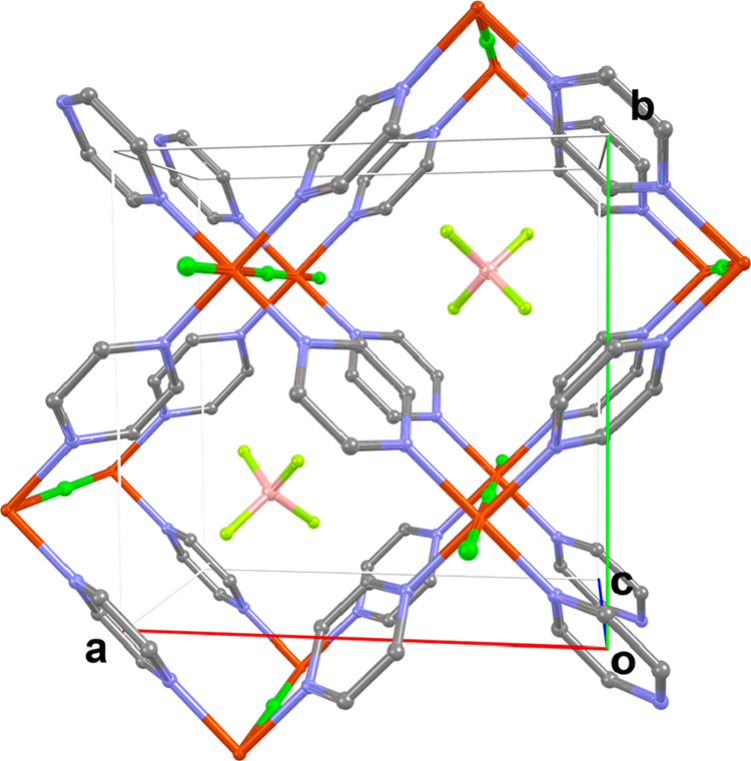
3D structure of the coordination polymers [CuX(pyz)_2_](BF_4_) with X = Cl or Br. Color code: orange for Cu, light green for X, gray for C, blue for N, pink for B, and yellow for F. Hydrogen atoms have been omitted for the sake of clarity.

This research focuses on the pressure-induced structural and magnetic changes of two of the most recent members of this family, [CuCl(pyz)_2_](BF_4_) and [CuBr(pyz)_2_](BF_4_)^12^ (Figure 1). They are ideal case studies because (a) they feature a crystallographic tetragonal symmetry, (b) the linker between two square mesh {[Cu(pyz)_2_]^2+^}_*n*_ layers is monatomic (Cl^–^ and Br^–^), and (c) the counterions are small and do not induce strain in the framework. Moreover, their crystal quality is excellent and, in fact, has allowed the accurate determination of their ground-state experimental electron charge density and correlated characterization of their chemical bonding properties in a previous work.^12^ Possible stereoelectronic responses to their compression include (a) strain of the covalent bonds in the framework linkers, (b) compression of the coordinative bonds to the metal ion, and (c) orbital reordering at the metal nodes, implying reorientation of the JT distortion axis. As a result, smooth and continuous or otherwise large and abrupt changes in the magnetic superexchange may occur. This means that the strength and pathway of the magnetic coupling can be modified, while the total spin state of the metals and the type of coupling are conserved.

We determined the pressure-dependent crystal structure of [CuCl(pyz)_2_](BF_4_) and [CuBr(pyz)_2_](BF_4_) up to ∼15 GPa and calculated the correlated effects on the magnetic couplings. While measurements of the magnetization at those pressures are not feasible yet, the accuracy of periodic calculations is established, and it allows prediction of the pressure-dependent interplay between the magnetic (sub)networks of this class of coordination polymers and therefore spurs experimental investigation of the magnetic properties under extreme conditions. Our study includes a chemical bonding analysis for the purpose of correlating the nature of the metal–linker interaction with the magnetic response.

## Experimental Section

### High-Pressure Single-Crystal X-ray Diffraction (HP SC-XRD)

**Figure 2:**
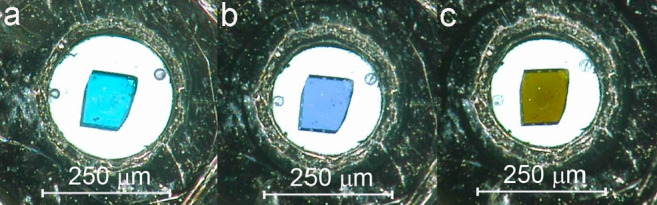
[CuCl(pyz)_2_](BF_4_) in DAC at (a) ∼0, (b) 10, and (c) 14 GPa.

Single crystals of [CuX(pyz)_2_](BF_4_) were loaded in Merrill-Bassett diamond-anvil cells (DACs)^19^ equipped with 0.5/0.6 mm diamonds and steel gaskets, preindented to ∼0.080/0.060 mm and with a 0.20/0.25 mm hole diameter (Figure 2). The experiments were repeated on a number of crystals using the same or different orientations of the sample with respect to the DAC body. These crystal loadings were labeled using progressive alphabetical letters from **a** to **e** in combination with the number **1** or **2** to refer to material [CuCl(pyz)_2_](BF_4_) or [CuBr(pyz)_2_](BF_4_), respectively. In loadings of samples **b**, **c**, and **e** of both coordination polymers, particular care was taken to orient the tetragonal axis of the crystal tilted in an oblique manner (/) or perpendicular (—) with respect to the DAC axis, in order to increase the completeness of data. Loading of sample **d** of both compounds was instead performed with the tetragonal crystallographic axis aligned parallel (|) with the DAC axis to explore the effects of the different orientation under nonhydrostatic conditions. A 4:1 (v/v) methanol/ethanol or 16:3:1 methanol/ethanol/water mixture was used as the pressure-transmitting medium. To improve hydrostaticity, the pressure variations above 10 GPa were applied after warming the DAC with a heat gun. The pressure was calibrated with the ruby fluorescence method.^20,21^ Single-crystal X-ray diffraction data were collected with an Oxford Diffraction SuperNova area-detector diffractometer using mirror optics monochromated Mo Kα radiation (λ = 0.71073 Å) or at the X04SA Material Science beamline of the Swiss Light Source (Paul Scherrer Institute)^22^ with a Pilatus 6M detector (λ = 0.49647 Å). CrysAlisPro^23^ was used for the data collection strategy, data reduction, and empirical absorption correction. The crystal structure for each pressure point was refined starting from the model of the previous pressure point with SHELXL-2014.^24^ Because of the high data quality and a sufficient data:parameter ratio, anisotropic thermal parameters could be refined for all atoms in measurements performed under the hydrostatic limit of 10 GPa. In most cases, only isotropic thermal parameters could be refined above this pressure. H atoms for the pyrazine ring were assigned geometrically and refined with a riding model with an isotropic thermal parameter equal to 1.2 times that of the corresponding parent atom. The results of these experiments are reported in Tables S1–S4.

### Density Functional Theory (DFT) Simulations

Starting from the structure determined at ambient pressure, the geometry of [CuX(pyz)_2_](BF_4_) with X = Cl or Br was optimized under hydrostatic pressure from 0 to 16 GPa with increasing steps of 2 GPa. The optimizations were done with the CRYSTAL14^25^ code in the tetragonal *P*4/*nbm* space group for all pressure points and additionally in the monoclinic *C*2/*m* space group only for pressures above 10 GPa. The unrestricted functional B3LYP with Grimme dispersion correction^26^ (scaling factor of 0.6) and the basis set pob-TZVP^27^ were used. The topology of the calculated electron density was analyzed with the AIMAll^28^ software through quantum theory of atoms in molecules (QTAIM).^29^The electron delocalization indices (DI) were also computed.

## Results and Discussion

### Compression under Quasi-hydrostatic Conditions

**Figure 3:**
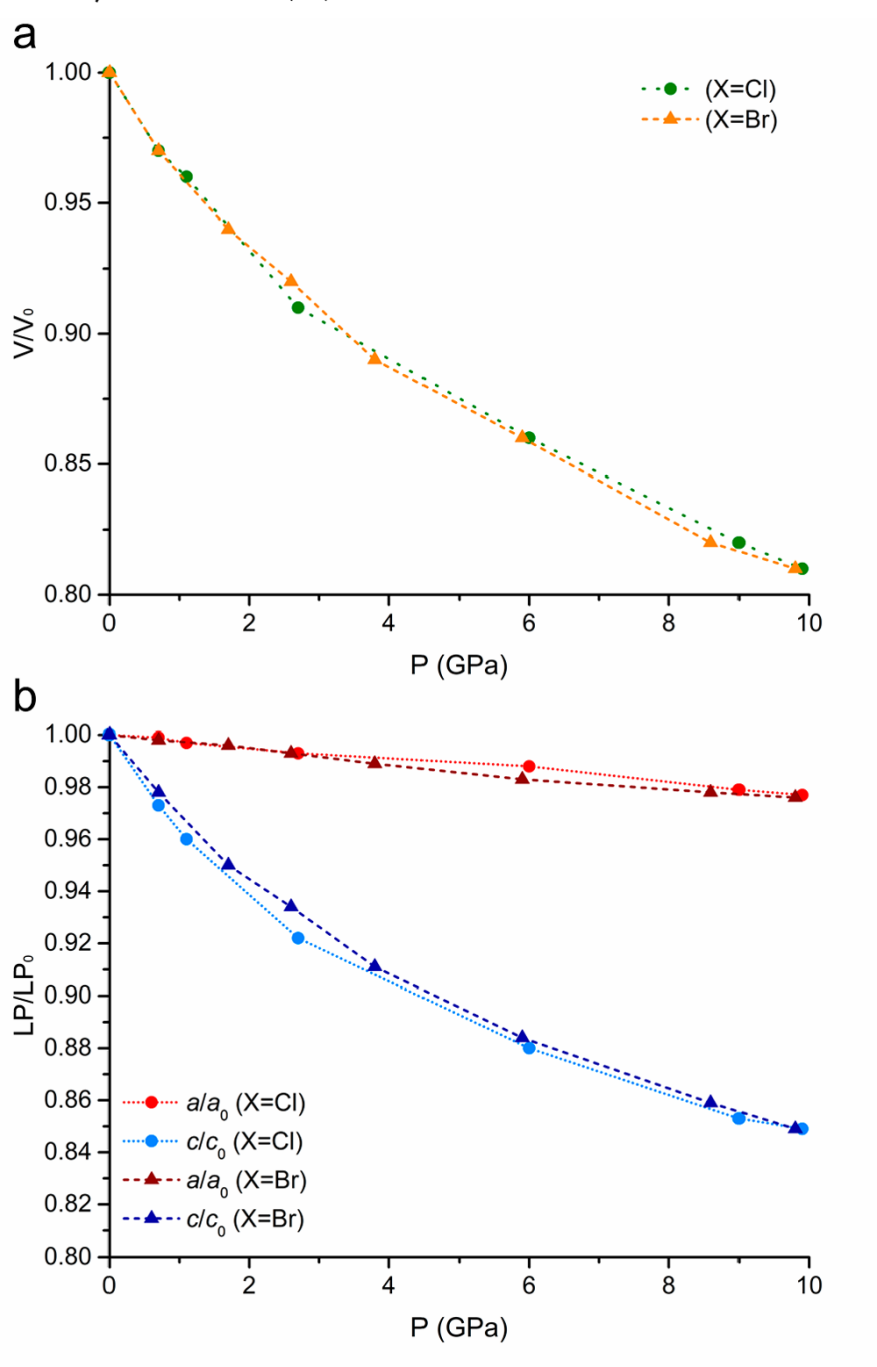
(a) Volumetric and (b) linear compressibility for [CuX(pyz)_2_](BF_4_) with X = Cl and Br under quasi-hydrostatic conditions of ≤10 GPa.

When [CuX(pyz)_2_](BF_4_) was compressed up to ∼10 GPa, a smooth shrinking of the crystal was observed, for the Cl and the Br derivatives. Their volumetric and linear relative compression is indeed very similar, showing a marked anisotropy (Figure 3). The most compressible direction is the *c* crystallographic axis, i.e., along the JT-distorted Cu–X bonds, while the deformation along the Cu−pyz bonds is minimal. Indeed, up to ∼10 GPa, the Cu–X distance decreases by ∼15%, while Cu–pyz by only ∼3%, which is on the same order of magnitude as the compression of the aromatic pyrazine skeleton. This behavior is completely reversible (see Figure S2).

The compressibility anisotropy resembles the thermal expansion behavior,^12^ with rigid {[Cu(pyz)_2_]^2+^}_*n*_ layers. The presence of a monatomic spacer and compact extra-framework counteranions allow the interlayer distance to be the smallest in the [CuX(pyz)_2_]Y family. Nevertheless, the interlayer connection is much more flexible, as typically observed for JT-distorted covalent bonds.^30^ With a high pressure, it is possible to force the Cu–X bond to approach its JT undistorted value. Under these extreme conditions, the stereoelectronic picture at the Cu^II^ ion can be significantly altered, with strong consequences on the magnetic properties of the crystal.

### Compression under Nonhydrostatic Conditions

Above ∼10 GPa, at room temperature, a glass transition of the pressure-transmitting medium makes the pressure nonhydrostatic. This leads to the generation of pressure gradients inside the DAC. A careful determination of the actual stress tensor is extremely difficult; however, it is reasonable to assume a quasi-uniaxial stress tensor aligned with the DAC axis. For experiments performed above the hydrostatic limit of the pressure-transmitting medium, a meticulous and clear description of the orientation of the crystal with respect to the main stress direction becomes necessary. Indeed, it is expected that bonds along the principal stress direction will be compressed more than under hydrostatic conditions, whereas the directions perpendicular to it will be less compressed or could even expand due to Poisson’s effect.^31^

**Figure 4:**
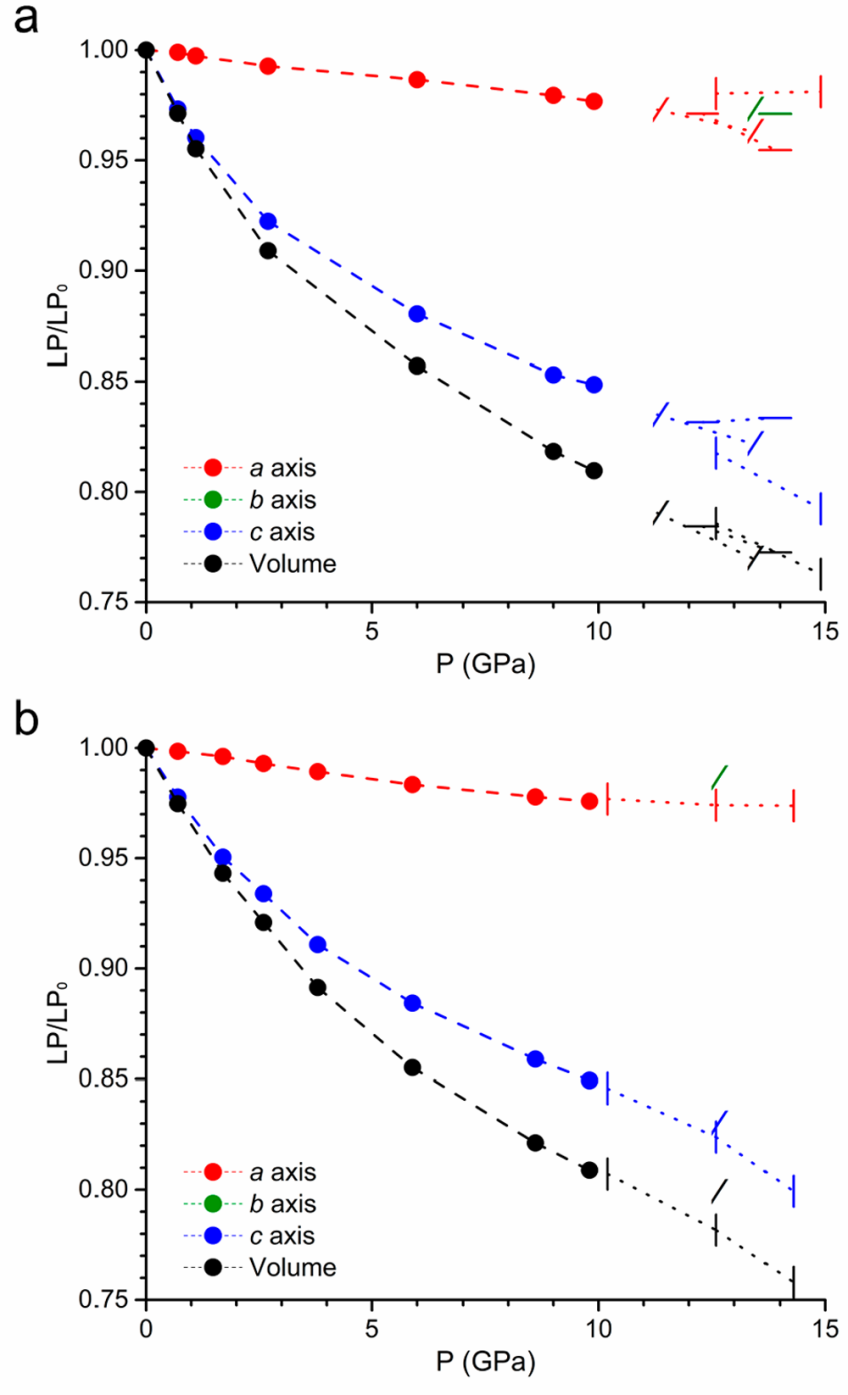
Volumetric and linear compressibility for [CuX(pyz)_2_](BF_4_) with (a) X = Cl and (b) X = Br at ≤15 GPa. Different symbols in the nonhydrostatic region (···) represent different DAC loadings with the *c* axis parallel (|), oblique (/), or orthogonal (—) with respect to the DAC axis. Note that crystallographic axes *a* and *b* are identical for quasi-hydrostatic compression (where the system remains tetragonal) but differ for oblique and orthogonal nonhydrostatic compression (breaking the tetragonal symmetry).

In fact, Figure 4 shows the cell parameters obtained from different DAC loadings, which clearly follow different trends above the quasi-hydrostatic limit, depending on the orientation of the crystal. Furthermore, when one of the equivalent *a* and *b* crystallographic axes is oriented along the main stress direction, the mechanical deformation leads to a dissymmetrization of the two axes, moving away from the tetragonal symmetry toward orthorhombic or monoclinic crystal systems.

**Figure 5:**
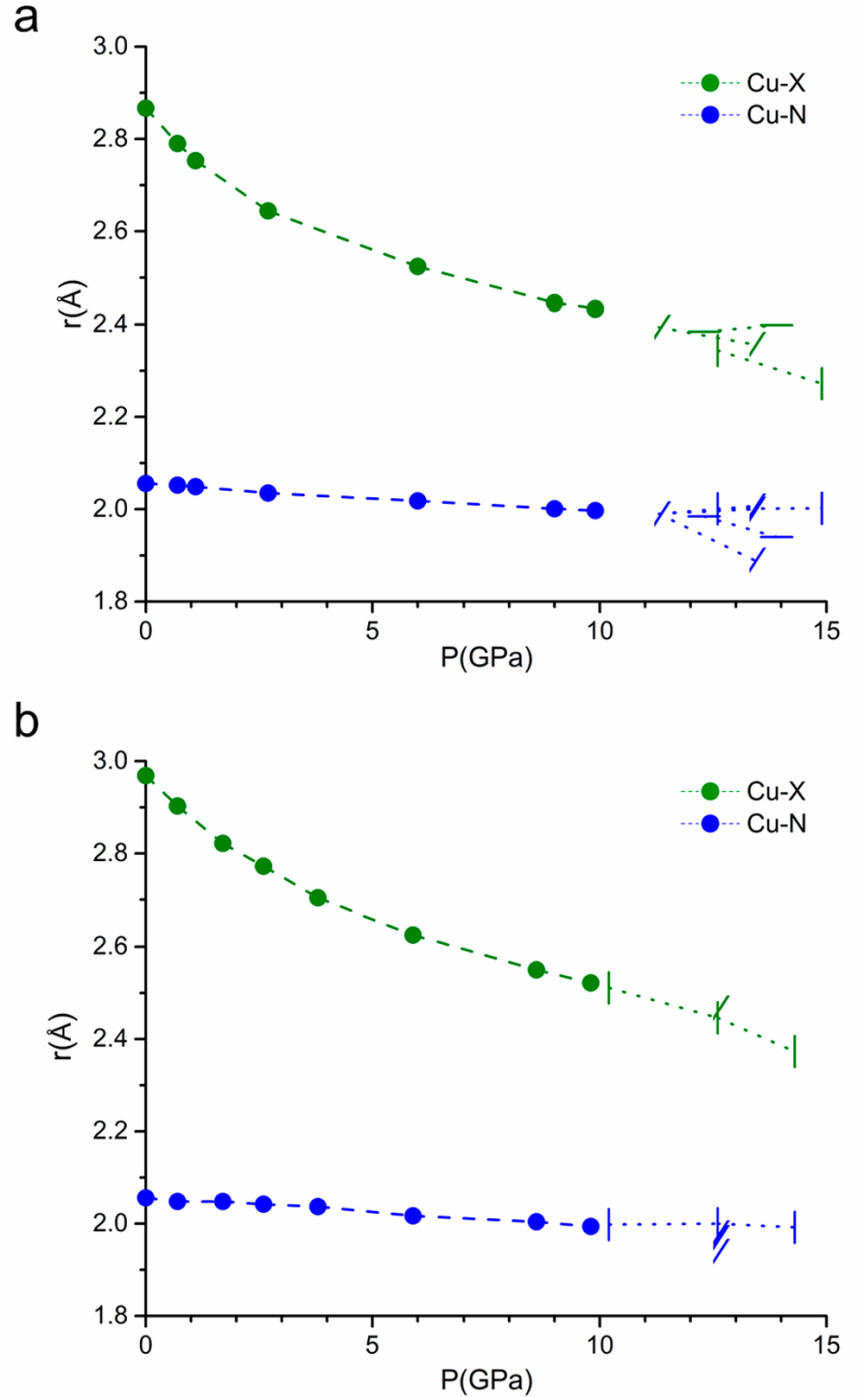
Cu–X and Cu–N distances for [CuX(pyz)_2_](BF_4_) with (a) X = Cl and (b) X = Br up to 15 GPa. Different symbols in the nonhydrostatic region (dotted lines) represent different DAC loadings with the *c* axis parallel (|), oblique (/), or orthogonal (—) with respect to the DAC axis.

For [CuCl(pyz)_2_](BF_4_), we collected three sets of data with different DAC loadings: sample **1c** (—) was loaded with the tetragonal axis perpendicular to the main stress direction, sample **1d** (|) with the same axis parallel to the main stress direction, and sample **1b** (/) in an intermediate oblique manner. For **1b** (/) loading (Figures 4a and 5a), we observed a fully reversible (Figure S2) phase transition at 13.4 GPa to the monoclinic *C*2/*m* space group, with a new doubly sized unit cell, the monoclinic axis oriented along the *a*–*b* diagonal of the parent tetragonal cell, and a monoclinic angle of 92.01(6)°. The transition causes dissymmetrization of the equatorial Cu–pyz bonds with small distortions of the angles accounting for the phase transition. Three of the pyz molecules surrounding the Cu center are no longer related by symmetry and have therefore different coordination distances (Figure 5a). Along the *a* axis, the bonds are shorter [1.89(2) Å], while the bond lengths along the monoclinic *b* axis are in line with those found at 11.3 GPa [2.007(10) and 2.000(10) Å compared to 1.990(3) Å at 11.3 GPa]. In the axial direction, no discontinuity is observed for the compressibility of the Cu–Cl bond [2.3570(8) Å], but a small deviation from the linearity of the chain is visible [Cu–Cl–Cu, 177.21(13)°]. As a consequence of the axial compression, the thickness of the {[Cu(pyz)_2_]^2+^}_*n*_ layers slightly decreases. In fact, the torsion angles between subsequent pyz rings along the chains increase with pressure, with a splitting at the phase transition. As seen for the distances, along the *b* axis the previous trend is followed, while along the *a* axis we observe a discontinuity and an increase in the torsion angle (Figure S3).

For sample **1c** (—) (Figures 4a and 5a), we observed instead a phase transition to orthorhombic space group *Cmma* at 13.9 GPa, probably due to the better alignment of a pyrazine chain with the principal stress direction that reduces the angular deformation. Here, two of the pyrazine molecules surrounding the Cu are not related by symmetry, and they both exhibit shorter distances with respect to that of the tetragonal structure at 12.3 GPa [1.9845(18) Å]. However, as expected, along the *a* axis (parallel to the DAC axis) we observe the shortest distance [1.94(3) vs 1.964(13) Å along the *b* axis]. Along the *c* axis, a slight elongation of the Cu–Cl bond from 2.3841(1) to 2.3987(9) Å is observed. In this case, any deviations from linearity of the framework chains are prohibited by symmetry. The splitting in the trend of the torsion angles between subsequent pyrazine molecules is more evident for this transition, with discontinuities in the increasing direction along the *a* axis, and in the decreasing one along the *b* axis (see Figure S3).

With regard to samples **1d** (|) (Figures 4a and 5a), oriented with the *c* axis parallel to the main stress direction, no phase transition is observed up to 14.9 GPa, because due to the orientation of the crystal, there is no dissymmetrization of the *a* and *b* axes. Not surprisingly, we observe the greatest shortening of the Cu–Cl bond [2.272(4) Å] and a significant elongation of the Cu–pyz bonds to 2.002(8) Å. We carefully checked the possible occurrence of one of the two phase transitions reported for samples **1b** (/) and **1c** (—), which would have generated a twinning, given the lowering of the symmetry from 4/*mmm* to 2/*m* or *mmm*. However, no splitting of peaks parallel to the *h*00 or 0*k*0 direction was observed. We also tested the possible occurrence of a lower-symmetry tetragonal phase, allowing for a −4 symmetry, compatible with an orbital reordering at Cu, alternating in the *a* or *b* direction. However, no symmetry decrease was detectable. Theoretical calculations (see below) also confirmed the genuine 4/*mmm* symmetry for this species, even when subjected to a uniaxial compression.

The behavior of samples **2c** (/) and **2d** (|) follows the considerations made for samples **1b** (/) and **1d** (|), respectively. A phase transition of sample **2e** (/) to the monoclinic *C*2*/m* space group at 12.6 GPa can be deduced from the unit cell parameters; however, the crystal quality was too low to extract a trustworthy geometric model of this phase. Attempts to model computationally the experimentally observed lower-symmetry phases always yielded exactly the same results of the calculations performed with the tetragonal geometry. This observation is in agreement with these phase transitions being mechanical deformation of the tetragonal geometry due to uniaxial stress but not being enthalpically stable in the hydrostatic regime. When one of the two equivalent axes is constrained to a shorter length (to simulate an increased stress in that direction), however, the calculation predicted an elongation of the other axis in line with our experimental observation.

### Simulations of the Structures and Prediction of the Magnetic Properties under Hydrostatic Compression

To exclude possible systematic errors in the experimental data, we optimized the experimentally determined geometries using periodic DFT at different pressures. Furthermore, this allowed us to investigate the pressure-induced electronic changes, which cannot be easily accessed from experimental data at HP. The computed structures showed a good agreement with the experiments and were used for the subsequent estimation of magnetic superexchange coupling constants (*J*) and analysis of the chemical bonds based on the electron charge density distribution.

At ambient pressure, as already presented in a previous study with magnetic susceptibility experiments and ambient-pressure experimental charge density analysis,^12^ the main superexchange pathway occurs through the pyrazine linkers with a σ-driven mechanism.^18^ The main reason for this to be the preferential pathway, despite the non-optimal efficiency of pyrazine as a superexchange linker, is that the halogen linkers lay along the pseudo-JT-distorted axis and are therefore orthogonal to the singularly occupied magnetic orbital. This results in an antiferromagnetic quasi-2D network, because *J*_pyz_ is ∼10 times larger than *J*_X_.

**Figure 6:**
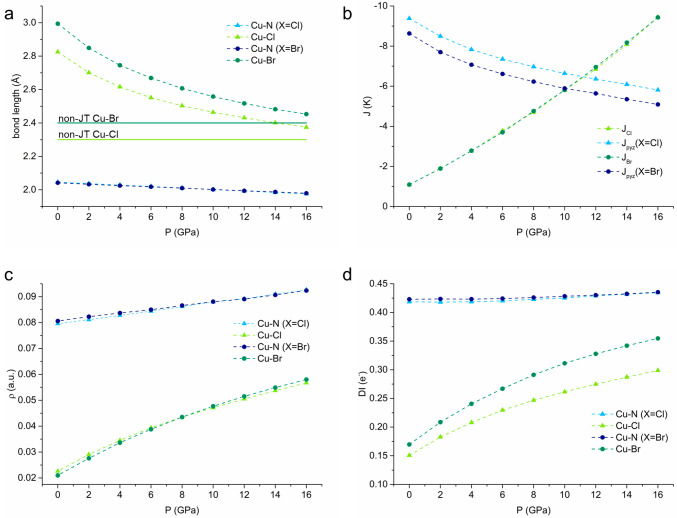
(a) Shortening of bond lengths with pressure. The dark and light green horizontal lines represent the ambient-pressure non-JT-distorted bond lengths for the Cu–Br and Cu–Cl bonds, respectively. (b) Magnetic superexchange coupling constants calculated with DFT in the gas phase as a function of pressure. Positive values of *J* correspond to antiferromagnetic coupling. (c and d) Topological analysis of the electron density. (c) ρ_bcp_ and (d) DI are the electron density at the bond critical point (e/bohr^3^) and the delocalization index (e^–^), respectively.

With pressure, both materials gradually switch from quasi-2D to 3D, due to an increase in the *J*_X_ at the expense of *J*_pyz_, until the two coupling constants become almost equivalent at around 10 GPa. Above this pressure, accompanying the fading of a clear JT-distorted direction (Figure 6a), the superexchange through the halogens becomes a better pathway, because of a combination of the short distance between Cu^II^ ions and the higher efficiency of a single-atom linker. It is noteworthy that the electronic state is unchanged, with the singly occupied d orbital of copper remaining the one perpendicular to the Cu–X bonds (typically addressed as *d*_*x*^2^-*y*^2^_) (see Table S7).

Interestingly, while structurally we observed that the main difference between the two materials is in the Cu–X distance, magnetically the situation is reversed. Indeed, the calculated *J*_Cl_ and *J*_Br_ are remarkably similar at each pressure point (see Figure 6b). On the other hand, *J*_pyz_ values of [CuCl(pyz)_2_](BF_4_) end up being ∼0.15 and ∼0.75 K larger than those of [CuBr(pyz)_2_](BF_4_) for periodic and gas phase DFT simulations, respectively (Table S5). This result is in good agreement with the experimental difference of ∼0.5 K obtained experimentally at ambient pressure.^12^ The two materials follow the same trend with an increase in pressure, maintaining the gap roughly constant (Figure 6b).

The almost pressure-independent Cu–pyz distances are mirrored by the electron density at the Cu–N bond critical points (ρ_bcp_) and the delocalization indices (DIs)^32^ that remain quite unchanged, despite the decrease in *J*_pyz_ described above as a function of pressure (Table S6). The increase in the electron density at the bond critical points of the Cu–X coordinative interactions with pressure correlates linearly with all of the observed changes in magnetic properties (Figure 6c). However, it does not address the reason why different *J*_pyz_’s are observed in the two compounds. In fact, the electron density at the Cu–X bond critical point coincides in the two materials. The DI of the Cu–X bond, which describes the covalent (open-shell) character of this interaction, increases also with an increase in pressure and the decrease in *J*_pyz_ as ρ_bcp_, but remarkably, it captures the difference between the two compounds at the same pressure (see Figure 6d). In particular, it is higher for the bond between Cu^II^ and Br^–^ than for the bond between Cu^II^ and Cl^–^ according to the softer nature of Br^–^ compared to Cl^–^. Indeed, upon withdrawal of spin density from the equatorial plane also shown by the increase in the overlap population in the Cu–X interaction as a function of pressure (Table S8), the larger DI of [CuBr(pyz)_2_](BF_4_) hampers superexchange through the pyrazine linkers with respect to the Cl analogue. This translates to the necessity of applying more pressure on [CuCl(pyz)_2_](BF_4_) compared to [CuBr(pyz)_2_](BF_4_) to achieve the same value of *J*_pyz_.

### Electrical Resistivity Measurements

The sample proved to be strongly insulating, both under ambient conditions and at high pressures of up to ∼5 GPa, indeed so much that it was not possible to measure a resistivity value because it fell outside of the measurable range of the instrument (see the Supporting Information for details about setup and measurement). We can therefore state only that the [CuCl(pyz)_2_](BF_4_) electrical resistivity should be ≥20 MΩ. This result agrees with the large band gap estimated from calculations of the structures as a function of pressure (Figure S4). The insulating nature of the sample makes it relevant for possible applications in spintronics. Indeed, insulating antiferromagnetic materials showed promising results for the development of spin-current generators^33−37^ and transmitters^38−42^ without involving charge transport.^4^

## Conclusions

In this work, we investigated the behavior of two isostructural electrical insulator antiferromagnetic coordination polymers, showing that their magnetic properties can be tuned using pressure as an external stimulus. The crystalline frameworks of [CuX(pyz)_2_](BF_4_) (X = Cl or Br) are remarkably stable at least up to ∼12.5 GPa. However, the computed magnetic properties highlight a pressure-induced switch from quasi-2D to 3D antiferromagnetic topology, and the stronger magnetic coupling occurs in the initially quasi-nonmagnetic direction, although the electronic state (and the magnetic orbital) of the metal atom remains the same. Such surprising behavior is justified by the better ability of monatomic X^–^ linkers to couple the spin active centers, especially compared to the pyrazine linkers. The coupling mechanism of pyrazine is in fact not efficient and therefore can be exceeded by that of the single-atom coupling groups.

The observed magnetic switch is induced by the shortening of the Cu–X bonds while the Cu–pyz distances remain almost constant. This means that the pseudo-Jahn–Teller distortion fades with an increase in pressure. Indeed, although under nonhydrostatic conditions, for both coordination polymers we reached a condition for which the Cu–X distances became comparable to those commonly observed for non-Jahn–Teller-distorted bonds at ambient pressure. This poses an interesting question regarding how the magnetic network would behave under these conditions. Theoretical calculations are possible but restricted to single-determinant wave functions (thus limited to a mixed state). More importantly, measurements of magnetization at these hydrostatic pressures are presently not possible: any progress in this direction may shed more light on a so far unexplored phenomenon. We brought into view the fact that Cu–X distances are reaching the limit of non-JT distortion, like the Cu–pyz ones, thus giving rise to a sort of “Jahn–Teller frustration”. We may envisage that many systems enter this regime, once pressurized, leading to an entirely new magnetic behavior, which can be switched through a mild external stimulus, and useful for spintronics applications.

## Supporting Information Available

Tables of crystallographic information, description of the electrical resistivity measures, graphs of the structural response under high pressure, and a table of the simulated structural and magnetic parameters and the calculated band gap (PDF)

## Accession Codes

CCDC 1999154–1999178 contain the supplementary crystallographic data for this paper. These data can be obtained free of charge via www.ccdc.cam.ac.uk/data_request/cif, or by emailing data_request@ccdc.cam.ac.uk, or by contacting The Cambridge Crystallographic Data Centre, 12 Union Road, Cambridge CB2 1EZ, UK; fax: +44 1223 336033.
