# Supplementary material for: Magnetic Network on Demand: Pressure Tunes Square Lattice Coordination Polymers Based on {[Cu(pyrazine)2]2+}n
Source: Inorg Chem. 2020 Jul 3;59(14):10091–8. doi: 10.1021/acs.inorgchem.0c01229 (PMC8008383; doi:10.1021/acs.inorgchem.0c01229)
Supplement: Supplementary file 1 — ic0c01229_si_001.pdf [file ic0c01229_si_001.pdf]

Magnetic network on demand: pressure tunes square lattice coordination polymers based on $\{[Cu(pyrazine)_2]^{2+}\}_n$.

Rebecca Scatena[a‡], Fabio Montisci[a¶*], Arianna Lanza[b], Nicola P. M. Casati[c], and Piero Macchi[a,d*]

[a] Department of Chemistry and Biochemistry, University of Bern, Freiestrasse 3, 3012 Bern, Switzerland

[b] Center for Nanotechnology Innovation @NEST, Istituto Italiano di Tecnologia, Piazza San Silvestro 12, 56127 Pisa, Italy

[c] Paul Scherrer Institute, Photon Science Division, WLGA/229 Forschungsstrasse 111, 5232 Villigen, Switzerland

[d] Department of Chemistry, Materials and Chemical Engineering, Polytechnic of Milan, via Mancinelli 7, 20131, Milan, Italy

Supporting Information

HP SC-XRD at different pressures

Table S1. Lattice parameters and structure refinement for $[CuCl(pyz)_2](BF_4)$ as a function of pressure at RT. For all entries: Formula = $C_8H_8BClCuF_4N_4$; *Mr* = 345.98g mol^{-1}; Symmetry = tetragonal, *P*4/*nbm*, *Z* = 2.

**Cl**, Sample	1a	1b	1b	1b
P /GPa	0.0001	0.7	1.1	2.7
unit cell dimensions				
a /Å	9.7547(1)	9.7448(3)	9.7288(5)	9.6838(4)
c /Å	5.7336(1)	5.5802(3)	5.5060(4)	5.2883(4)
V /Å^3	545.58(2)	529.90(4)	521.14(7)	495.92(6)
reflections (unique)	4326 (525)	2629 (269)	1988 (269)	1847 (253)
R_{int}	0.0198	0.0383	0.0418	0.0477
parameters (restraints)	28 (0)	27 (0)	27 (0)	27 (0)
goodness-of-fit on F^2	1.077	1.068	1.095	1.058
R_1 for $I > 2\sigma(I)$, all	0.0241, 0.0266	0.0268, 0.0401	0.0262, 0.0487	0.0294, 0.0529
wR_2 for $I > 2\sigma(I)$, all	0.0607, 0.0624	0.0552, 0.0604	0.0457, 0.0530	0.0627, 0.0776
largest residues /e Å^{-3}	0.52 and -0.29	0.25 and −0.27	0.34 and −0.51	0.50 and −0.59

**Cl** *continued*, Sample	1b	1b	1c
P /GPa	6.0	9.0	9.9
unit cell dimensions			
a /Å	9.6240(3)	9.5543(4)	9.5280(4)
c /Å	5.0476(3)	4.8911(4)	4.8655(2)
V /Å^3	467.52(4)	446.48(5)	441.70(4)
reflections (unique)	1746 (240)	1670 (232)	1549(158)
R_{int}	0.0467	0.0464	0.0420
parameters (restraints)	27 (0)	27 (0)	27(0)
goodness-of-fit on F^2	1.086	1.089	1.150
R_1 for $I > 2\sigma(I)$, all	0.0287, 0.0517	0.0323, 0.0630	0.0208, 0.0276
wR_2 for $I > 2\sigma(I)$, all	0.0619, 0.0687	0.0633, 0.0768	0.0456, 0.0479
largest residues /e Å^{-3}	0.35 and -0.52	0.697 and −0.56	0.22 and −0.39

Table S2. Lattice parameters and structure refinement for $[CuBr(pyz)_2](BF_4)$ as a function of pressure at RT. For all entries: Formula = $C_8H_8BBrCuF_4N_4$; *Mr* = 390.45g mol^{-1}; Symmetry = tetragonal, *P*4/*nbm*, *Z* = 2.

**Br**, Sample	2a	2b	2b	2e (—)
P /GPa	0.0001	0.7	1.7	2.6
unit cell dimensions				
a/Å	9.7651(2)	9.7500(7)	9.7275(6)	9.6964(3)
c/Å	5.9377(2)	5.8056(5)	5.6437(5)	5.5452(3)
V /Å^3	566.20(3)	551.89(9)	534.03(8)	521.36(4)
reflections (unique)	1753 (357)	2106 (239)	2073 (231)	2042 (251)
R_{int}	0.0145	0.0577	0.0667	0.0279
parameters (restraints)	27 (0)	27 (0)	27 (0)	27 (0)
goodness-of-fit on F^2	1.053	1.058	1.040	1.075
R_1 for $I > 2\sigma(I)$, all	0.0200, 0.0228	0.0295, 0.0622	0.0305, 0.0724	0.0213, 0.0382
wR_2 for $I > 2\sigma(I)$, all	0.0498, 0.0519	0.0535, 0.0635	0.0622, 0.0761	0.0438, 0.0489
largest residues /e Å^{-3}	0.37 and −0.40	0.414 and −0.33	0.44 and −0.34	0.30 and −0.25

**Br** *continued*, Sample	2b	2e (—)	2c	2e (—)
P /GPa	3.8	5.9	8.6	9.8
unit cell dimensions				
a/Å	9.6603(11)	9.6027(7)	9.5485(7)	9.5291(9)
c /Å	5.4081(9)	5.2504(6)	5.0998(17)	5.0433(7)
V /Å^3	504.69(14)	484.15(9)	464.97(17)	457.95(11)
reflections (unique)	1894 (221)	1832 (235)	1630(201)	1673 (214)
R_{int}	0.0709	0.0355	0.0370	0.0546
parameters (restraints)	27 (0)	27 (0)	27 (0)	27 (0)
goodness-of-fit on F^2	1.068	1.055	1.171	1.062
R_1 for $I > 2\sigma(I)$, all	0.0408, 0.0769	0.0253, 0.0516	0.0278, 0.0403	0.0415, 0.0711
wR_2 for $I > 2\sigma(I)$, all	0.0838, 0.0974	0.0510, 0.0587	0.0643, 0.0695	0.0948, 0.1104
largest residues /e Å^{-3}	1.30 and −0.39	0.82 and −0.36	0.44 and −0.28	0.77 and −0.52

Table S3. Lattice parameters and structure refinement for $[CuCl(pyz)_2](BF_4)$ in non-hydrostatic pressure range. For all entries: Formula = $C_8H_8BClCuF_4N_4$; *Mr* = 345.98g mol^{-1}.

**Cl** *non-hydrostatic*, Sample	1b (/)	1c (—)	1d (|)
P /GPa	11.3	12.3	12.6
Symmetry	Tetragonal	Tetragonal	Tetragonal
Space Group, Z	*P4/nbm*, 2	*P4/nbm, 2*	*P4/nbm, 2*
unit cell dimensions			
a/Å	9.4908(4)	9.4738(2)	9.5625(10)
b/Å			
c /Å	4.7877(4)	4.7682(1)	4.688(6)
β/°			
V /Å^3	431.25(5)	427.96(2)	428.6(6)
reflections (unique)	1606 (216)	4066 (350)	2376 (115)
R_{int}	0.0436	0.0454	0.0814
parameters (restraints)	27 (0)	27 (0)	13 (0)
goodness-of-fit on F^2	1.057	1.093	1.206
R_1 for $I > 2\sigma(I)$, all	0.0314, 0.0486	0.0318, 0.0420	0.0733, 0.0752
wR_2 for $I > 2\sigma(I)$, all	0.0686, 0.0768	0.0837, 0.0886	0.1587, 0.1605
largest residues /e Å^{-3}	0.38 and -0.41	0.57 and -0.42	1.90 and -0.52

**Cl** *non-hydrostatic continued*, Sample	1b (/)	1c (—)	1d (|)
P /GPa	13.4	13.9	14.9
Symmetry	Monoclinic	Orthorhombic	Tetragonal
Space Group, Z	*C2/m*, 4	*Cmma, 4*	*P4/nbm, 2*
unit cell dimensions			
a/Å	13.287(13)	13.17(3)	9.5708(16)
b/Å	13.4257(10)	13.397(3)	
c /Å	4.7126(15)	4.7793(17)	4.543(8)
β/°	92.01(6)		
V /Å^3	840.1(9)	844(2)	416.1(7)
reflections (unique)	2047 (281)	2538 (214)	2380 (115)
R_{int}	0.0599	0.0438	0.1802
parameters (restraints)	42 (0)	23 (0)	13 (0)
goodness-of-fit on F^2	1.077	1.265	1.358
R_1 for $I > 2\sigma(I)$, all	0.0551, 0.0841	0.1111, 0.1174	0.1187, 0.1328
wR_2 for $I > 2\sigma(I)$, all	0.1437, 0.1656	0.2918, 0.2982	0.2615, 0.2874
largest residues /e Å^{-3}	0.42 and -0.57	1.33 and -1.27	3.18 and -0.82

Table S4. Lattice parameters and structure refinement for $[CuBr(pyz)_2](BF_4)$ in non-hydrostatic pressure range. For all entries: Formula = $C_8H_8BBrCuF_4N_4$; *Mr* = 390.45g mol^{-1}.

**Br** *non-hydrostatic*, Sample	2d (|)	2d (|)	2c (/)	2d (|)
P /GPa	10.2	12.6	12.6	14.3
Symmetry	Tetragonal	Tetragonal	Monoclinic	Tetragonal
Space Group, Z	*P4/nbm, 2*	*P4/nbm, 2*	*C2/m, 4*	*P4/nbm, 2*
unit cell dimensions				
a/Å	9.5391(3)	9.5119(3)	13.48(4)	9.5092(4)
b/Å			13.623(8)	
c /Å	5.022(4)	4.892(4)	4.93(2)	4.746(5)
β/°			91.6(4)	
V /Å^3	457.0(3)	442.6(4)	906(5)	429.2(4)
reflections (unique)	2059 (99)	1802 (94)	1026 (194)	1911 (97)
R_{int}	0.0932	0.0822	0.2714	0.1045
parameters (restraints)	13 (0)	13 (0)	42 (45)	13 (0)
goodness-of-fit on F^2	1.243	1.142	1.067	1.123
R_1 for $I > 2\sigma(I)$, all	0.0417, 0.0491	0.0344, 0.0419	0.1417 , 0.2100	0.045, 0.0517
wR_2 for $I > 2\sigma(I)$, all	0.1214, 0.1289	0.0985, 0.1047	0.3159, 0.3714	0.1146, 0.1214
largest residues /e Å^{-3}	0.47 and -0.43	0.39 and -0.32	0.60 and -0.57	0.41 and -0.37

Electrical Resistivity Measurements

The electrical resistivity of several single crystals of $[CuCl(pyz)_2](BF_4)$ with ~0.3 × 0.3 × 0.1 mm size was measured at ambient conditions with the four-probes method and a Keithley 2450 multimeter (Tektronix); the four golden leads where attached to the sample using Silver-Epoxy glue.

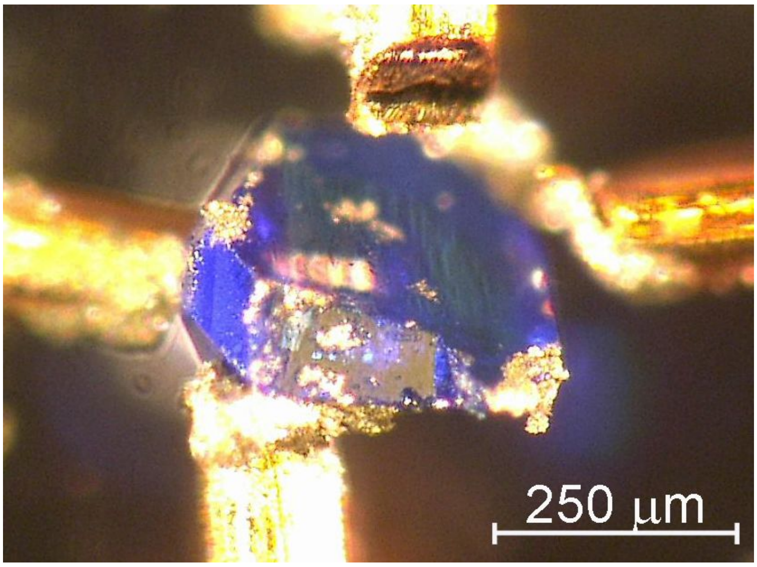

Figure S1. Setup for electrical resistivity measurement at ambient pressure.

A smaller crystal (~0.15 × 0.12 × 0.06 mm) was then loaded in a Merrill-Bassett DAC [18] for measurements at high pressure; two golden wires were pressed against the sample and the electrical resistivity was measured using the two-probe method and a Keithley 2450 multimeter (Tektronix) up to ~5GPa. A mixture of α-nanoalumina and epoxy glue 4:1 was employed as pressure transmitting medium, and the ruby fluorescence method [19] [20] was used for pressure calibration.

Structural response of [CuCl(pyz)$_2$](BF$_4$) under pressure

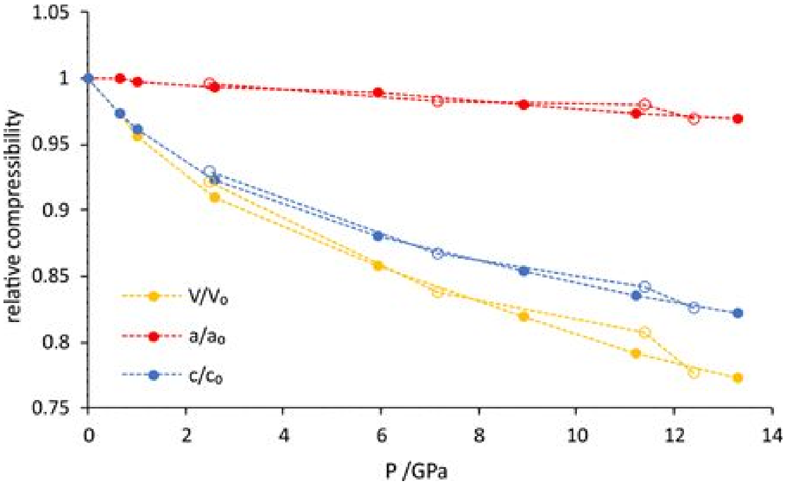

Figure S2. Elastic behavior of sample b of [CuCl(pyz)$_2$](BF$_4$). The open symbols indicate points measured during decompression and show full reversibility of the compression.

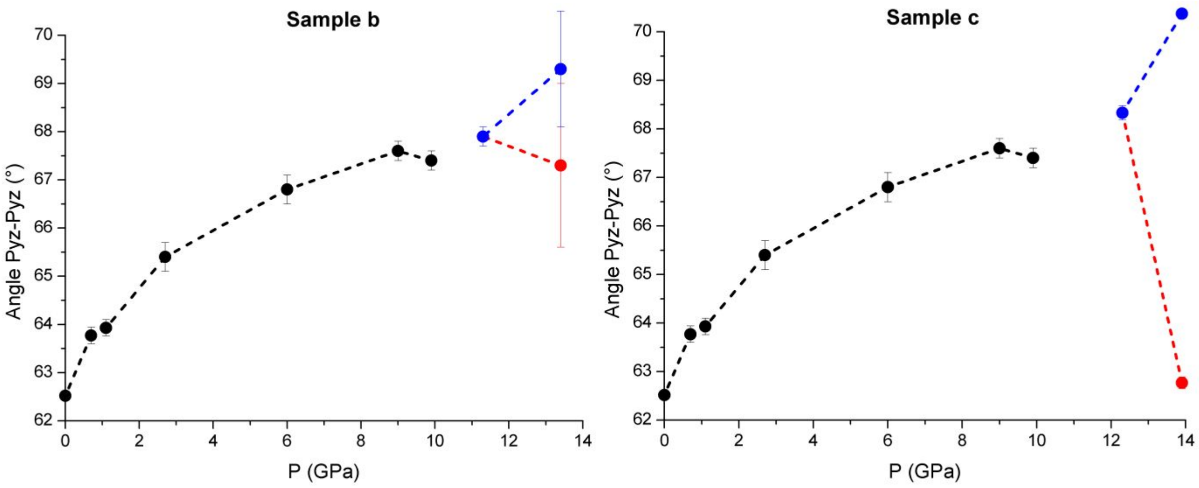

Figure S3. Torsion angle between adjacent pyrazines along the chains for samples b (left) and c (right).

Simulations of structure and magnetic properties under hydrostatic compression

Table S5. Calculated magnetic super-exchange coupling constants for $[CuX(pyz)_2](BF_4)$ as a function of pressure in periodic-DFT (p-DFT) and gas phase DFT (gp-DFT). Positive values of J correspond to antiferromagnetic coupling.

Pressure (GPa)	$[CuCl(pyz)_2](BF_4)$				$[CuBr(pyz)_2](BF_4)$			
	J_{pyz} (K)		J_{Cl} (K)		J_{pyz} (K)		J_{Br} (K)	
	p-DFT	gp-DFT	p-DFT	gp-DFT	p-DFT	gp-DFT	p-DFT	gp-DFT
0	8.46	9.38	0.91	1.09	8.36	8.63	0.82	1.09
2	7.53	8.49	1.42	1.90	7.31	7.70	1.39	1.89
4	6.80	7.83	2.04	2.77	6.62	7.07	2.01	2.78
6	6.35	7.35	2.76	3.78	6.22	6.61	2.67	3.70
8	5.86	6.97	3.48	4.70	5.75	6.23	3.45	4.76
10	5.47	6.64	4.31	5.81	5.29	5.89	4.32	5.81
12	5.16	6.36	5.24	6.85	4.99	5.64	5.17	6.95
14	4.83	6.09	6.25	8.08	4.65	5.35	6.12	8.17
16	4.43	5.81	7.40	9.44	4.32	5.09	7.14	9.43

Table S6. Topological analysis of the electron density. ρ_{bcp} and DI are respectively the electron density at the bond critical point ($e/bohr^3$) and the delocalization index (e^-).

Pressure (GPa)	$[CuCl(pyz)_2](BF_4)$				$[CuBr(pyz)_2](BF_4)$			
	Cu—pyz		Cu—Cl		Cu—pyz		Cu—Br	
	ρ_{bcp}	DI	ρ_{bcp}	DI	ρ_{bcp}	DI	ρ_{bcp}	DI
0	0.0796	0.4186	0.0226	0.1506	0.0806	0.4231	0.0210	0.1697
2	0.0811	0.4180	0.0291	0.1827	0.0823	0.4235	0.0276	0.2086
4	0.0828	0.4186	0.0345	0.2078	0.0837	0.4232	0.0336	0.2405
6	0.0844	0.4202	0.0394	0.2295	0.085	0.4242	0.0388	0.2669
8	0.0862	0.4228	0.0435	0.2468	0.0866	0.4260	0.0435	0.2911
10	0.0880	0.4252	0.0471	0.2614	0.0881	0.4283	0.0477	0.3112
12	0.0892	0.4290	0.0505	0.2748	0.0891	0.4302	0.0515	0.3277
14	0.0910	0.4318	0.0537	0.2871	0.0907	0.4324	0.0549	0.3419
16	0.0927	0.4345	0.0567	0.2988	0.0924	0.4354	0.0580	0.3546

Table S7. d-orbital population od the Cu node as a function of pressure.

d-orbital	0 GPa	2 GPa	4 GPa	6 GPa	8 GPa	10 GPa	12 GPa	14 GPa	16 GPa
[CuCl(pyz)2](BF4)									
z^2	1.932	1.935	1.935	1.938	1.939	1.939	1.939	1.939	1.940
xz	1.934	1.933	1.930	1.929	1.928	1.926	1.925	1.924	1.922
yz	1.934	1.933	1.930	1.929	1.928	1.926	1.925	1.924	1.922
xy	1.935	1.932	1.929	1.928	1.926	1.925	1.922	1.921	1.920
x^2-y^2	1.293	1.291	1.292	1.292	1.293	1.295	1.297	1.300	1.301
[CuBr(pyz)2](BF4)									
z^2	1.926	1.928	1.930	1.932	1.933	1.933	1.934	1.935	1.935
xz	1.933	1.932	1.930	1.929	1.927	1.925	1.923	1.921	1.921
yz	1.933	1.932	1.930	1.929	1.927	1.925	1.923	1.921	1.921
xy	1.934	1.931	1.929	1.928	1.925	1.924	1.922	1.920	1.920
x^2-y^2	1.299	1.297	1.297	1.299	1.300	1.302	1.304	1.309	1.309

Table S8. Overlap population (e^-) obtained from the Mulliken population analysis for selected interactions as a function of pressure.

interaction	0 GPa	2 GPa	4 GPa	6 GPa	8 GPa	10 GPa	12 GPa	14 GPa	16 GPa
[CuCl(pyz)2](BF4)									
Cu-N	0.076	0.077	0.077	0.076	0.074	0.074	0.073	0.071	0.069
Cu-Cl	0.022	0.026	0.029	0.032	0.033	0.036	0.039	0.041	0.042
Cu-C	-0.005	-0.005	-0.006	-0.006	-0.006	-0.006	-0.006	-0.006	-0.006
[CuBr(pyz)2](BF4)									
Cu-N	0.076	0.077	0.076	0.076	0.075	0.074	0.073	0.072	0.071
Cu-Br	0.018	0.021	0.024	0.025	0.026	0.028	0.027	0.028	0.03
Cu-C	-0.006	-0.006	-0.006	-0.006	-0.006	-0.007	-0.007	-0.007	-0.007

Calculated band gap

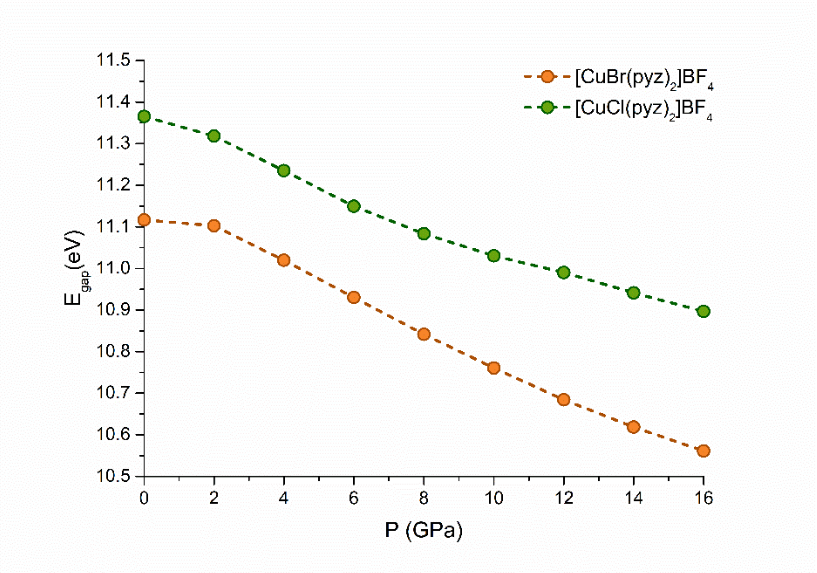

Figure S4. Band gap for calculated structures of $[CuBr(pyz)_2](BF_4)$ and $[CuCl(pyz)_2](BF_4)$ up to 16GPa.
